# Genetic analysis of four European huchen (*Hucho hucho* Linnaeus, 1758) broodstocks from Poland, Germany, Slovakia, and Ukraine: implication for conservation

**DOI:** 10.1007/s13353-015-0274-9

**Published:** 2015-03-06

**Authors:** M. Kucinski, D. Fopp-Bayat, T. Liszewski, V. W. Svinger, I. Lebeda, R. Kolman

**Affiliations:** 1 Department of Ichthyology, University of Warmia and Mazury in Olsztyn, ul. Oczapowskiego 5, 10-719 Olsztyn, Poland; 2 Fachberatung für Fischerei des Bezirks Oberfranken, Ludwigstraße 20, 95444 Bayreuth, Germany; 3 Faculty of Fisheries and Protection of Waters, University of South Bohemia in České Budějovice, 38925 Vodňany, Czech Republic; 4 Department of Ichthyology, Inland Fisheries Institute in Olsztyn, 10-718 Olsztyn, Kortowo Poland

**Keywords:** Broodstocks, Conservation, Genetic distance, Genetic diversity, *Hucho hucho*, Microsatellite DNA

## Abstract

Four broodstocks of European huchen (*Hucho hucho*) from: Poland, Germany, Slovakia, and Ukraine were investigated using ten microsatellite DNA loci. Microsatellite DNA analysis was successfully applied for the first time in the Polish broodstock of this fish species. The genetic variation and genetic distance between these broodstocks were evaluated. In addition, we examined the potential effects of a genetic bottleneck on the genetic variation of the broodstocks. The European huchen broodstocks exhibited moderate genetic diversity (*PIC* = 0.405–0.496 and *I* = 0.831–1.047) with the exception of German broodstock which presented higher genetic diversity (*PIC* = 0.590 and *I* = 1.254). Observed (*Ho*) and expected (*He*) heterozygosity across the investigated loci in all broodstocks ranged from 0.434 to 0.686 and from 0.452 to 0.650, respectively. Overall, the studied broodstocks were in Hardy-Weinberg equilibrium (HWE); however, from 8 to 42 % of the loci deviated from HWE in each stock. The Garza-Williamson index (*M* = 0.146–0.279) and values of the heterozygosity excess revealed a reduction of genetic variation in all studied broodstocks because of the founder or bottleneck effect. The analysis of genetic differentiation (*Fst*) and Nei’s genetic distance between pairs of broodstocks revealed that Polish and Ukrainian broodstocks of European huchen were characterized by the closest genetic distance. In contrast, the highest genetic divergence parameters (*Fst* and Nei’s distance) were observed among German, Slovak, and Ukrainian broodstocks.

## Introduction

European huchen (*Hucho hucho*), also called Danube salmon, is the largest salmonid species and one of the most endangered members of the Salmonidae family (Holcik 1995). It is endemic to the Danube drainage in Central Europe, occurring in cool montane and submontane reaches of large streams and swift rivers (Kottelat and Freyhof 2007). European huchen, together with its sister species taimen (*Hucho taimen*) are exclusively freshwater residents. Furthermore, this fish species can live for more than 20 years, and adults are large piscivorous predators of up to 1.65 m standard length (SL) and with a maximum mass of 60 kg (Kottelat and Freyhof 2007). European huchen is a good indicator for overall stream connectivity and ecosystem health due to the species’ stringent water and substratum quality requirements as well as its facultative migratory spawning behavior. European huchen is listed in annex II and annex V of the European Fauna Flora Habitat (FFH) directive 92/43/EWG and is considered as an endangered species with respect to its global distribution (IUCN 2013), including Poland (PRDBoA 2002). In recent years the size of the European huchen populations has strongly declined through the effects of anthropogenic habitat alterations, such as: river regulation by constructing of dams and weirs, siltation of spawning ground, hydropower development as well as both industrial and agricultural pollution (Holcik 1990; Witkowski et al. 2013a). In Austria, European huchen inhabit 10 % of their previous distribution, considering self-sustaining populations (Schmutz et al. 2002). Distinctive characters of the species make European huchen a popular target species for anglers and a flagship species for conservation of running waters (Geist et al. 2009). European huchen are bred in artificial conditions for a fishery supplementation of the local populations. According to some authors, due to low natural reproduction success most of the wild populations of the European huchen depend on the stocking activities (Holcik 1995; Witkowski et al. 2013a). In Poland European huchen occurs in Dunajec, Poprad, and San Rivers, where it is protected by a fishery supplementation and enacted rigorous fishing restrictions. At present, there is only one fish farm, situated in Lopuszna (Southern Poland) producing between 800 thousand to 1 million individuals of hatch annually, which is the only available source for European huchen stocking material in Poland (Witkowski et al. 2013b). Furthermore, available data suggests that hatchery stock of European huchen in Lopuszna (Poland) was established by small number of captured spawners. In 1955 only six (three females and three males) and in 1963 another few fish were caught from Czarna Orava River. Additionally, in 1985, this stock was only once supplemented by 30 females originated from Slovakia (Witkowski et al. 2013b).

Genetic data on the European huchen are still sparse and mainly limited to phylogenetic analysis of a few individuals in higher order systematic studies (e.g., Phillips et al. 1995; Crespi and Fulton 2004) or research focused on the related taxa, such as: taimen (Froufe et al. 2005; Guangxiang et al. 2006; You-Yi et al. 2009; Liu et al. 2011), lenok (*Brachymystax lenok*) (Xia et al. 2006) and Sichuan taimen (*Hucho bleekeri*) (Wang et al. 2011). Recently, a conservation genetic study on geographically limited European huchen populations from Austria, Slovenia, Bosnia-Herzogevenia, Montenegro, Germany, Slovakia, and Ukraine has been performed, which provided some fragmentary information about their genetic structure and variability (Geist et al. 2009; Weiss et al. 2011). Although information on the genetic diversity and differentiation is urgently needed for the conservation management of European huchen these data are still unavailable for the Polish population of this fish species. Additionally, there are many studies that demonstrate the benefits of conservation management based on genetic data (Hansen 2002; Gum et al. 2003, 2006; Fopp-Bayat 2010; Olsson et al. 2012). Thus, studies on the genetic structure of stock or population are important during conservation management for endangered fish species being protected by a rehabilitation measure, such as European huchen.

The major objectives of the present study were assessment of the current genetic diversity of European huchen broodstock in Poland by means of microsatellite DNA analysis and comparison of it with samples from German, Slovak and Ukrainian broodstocks. The results of the research will provide baseline data for the improvement of existing conservation program management of the European huchen broodstock in Poland.

## Materials and methods

### Sample collection and DNA extraction

**Fig. 1:**
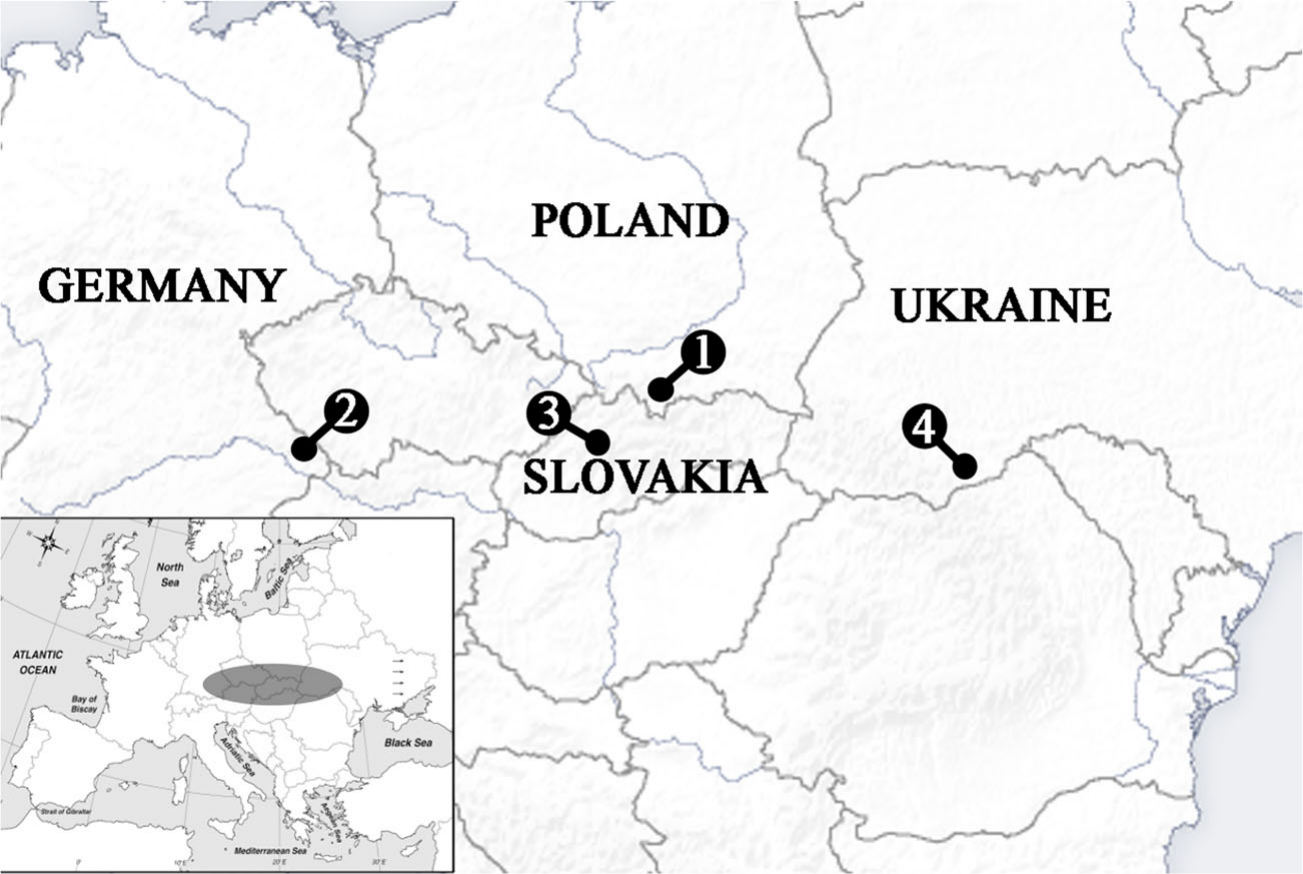
Sampling locations of European huchen: 1. Restocking Centre and Trout Hatchery Lopuszna, Poland; 2. Fish farm Lindbergmuehle, Bavaria, Germany; 3. Fish farm Pribovce, Martin Province, Slovakia; 4. Fish farm “Ishkhan” Baniliv, Chernivtsi Province, Ukraine

Fin clips from a total of 135 European huchen specimens were non-invasively sampled from four European huchen broodstocks and utilized for the genetic analysis. Fish tissues originated from fish farms localized in: Poland (Restocking Centre and Trout Hatchery Lopuszna), Germany (Fish farm Lindbergmuehle, Bavaria), Slovakia (Fish farm Pribovce, Martin Province) and Ukraine (Fish farm “Ishkhan” Baniliv, Chemivtsi Province) during the years 2011-2013 (Fig. 1). Small (<1 cm^2^) pelvic or pectoral fin clips were placed in Eppendorf tubes and kept in 96 % ethanol at a temperature of 4 °C until DNA extraction. DNA was isolated from collected fin clips using standard Chelex 100 procedure (Walsh et al. 1991).

### Polymerase chain reaction amplification (PCR)

A total of 29 microsatellite fragments, previously established for salmonids: *BleTet-2, BleTet-9, BleTri-2* (Froufe et al. 2004), *Ssa-171, Ssa-197, Ssa-85* (O’Reilly et al. 1996), *Str-543, Str-85* (Presa and Guymard 1996), *Str-12* (Poteaux et al. 1999), *Sfo-292, Sfo-262* (Perry et al. 2005), *Str-15, Str-60* (Sonstebo et al. 2007), *Str-73* (Estoup et al. 1993), *Ogo-2* (Olsen et al. 1998), *Sfo-18* (Geist et al. 2009), *Hljz-003, Hljz-023* (Guangxiang et al. 2006), *Omm-1016, Omm-1032, Omm-1077, Omm-1088, Omm-1097, Omm-1125, Omm-5000, Omm-5017, Hljz-031, Hljz-056* as well as *Hljz-069* (You-Yi et al. 2009) were tested for cross-amplification in four broodstocks of European huchen. Reaction mixtures were prepared in a total volume of 25 μl with a 0.8 μl DNA template (4.6 ± 0.5 μg/ml), 5.0 μl of 10× PCR reaction buffer (100 mM Tris-HCl pH 9.0, 500 mM KCl, Triton X-100), 0.5 μl of each primer, 0.5 μl (500 μM) of each deoxynucleotide triphosphate (dNTP), 0.8-1.0 μl MgCl_2_ (25 mM/μl) and 0.6 unit GoTaq flexi DNA polymerase (Lucigen, Middleton, WI, USA). Re-distilled water was used to bring the reaction mixture to the desired final volume. Amplification was performed with a Mastercycler gradient thermocycler (Eppendorf, Germany) under the following conditions: an initial denaturation at 94 °C for 3 min, followed by 34 cycles at 94 °C for 30 s, annealing at 53–61 °C (Table 1) for 45 s, elongation at 72 °C for 45 s and a final elongation step at 72 °C for 7 to 10 min.

**Table 1:** Characterization of ten microsatellite loci applied in study of European huchen: locus designation, primer sequences, optimal annealing temperature (*T*_a_), MgCl_2_ concentration, number of observed alleles (*A*_o_), allele size range, fluorescence dye (Dye) used for detection on the ABI 3130 and source reference. Tetrasomic loci were underlined

Locus	Primer sequence (5’ → 3’)	*T*_a_ (°C)	MgCl_2_ (mM)	*A*_o_	Allele size range (bp)	Dye	Reference
*BleTet-9*	F: ACTGGATAGAAAGACCTGTGG	53	0.8	15	281–422	Ned	Froufe et al. 2004
	R: AGATTCTTGGTAAAAGTGAAG						
*BleTri-2*	F: CCAGGACATATTCCCTTCTAG	55	0.8	3	118–127	Ned	Froufe et al. 2004
	R: CCACAGCTCAGGGCAGGGAGT						
*Hljz-056*	F: CTCTGTCTCATCTCGCTT	57	0.8	4	224–236	Pet	You-Yi et al. 2009
	R: TTCACTTGGTGTAATGGC						
*Ogo-2*	F: ACATCGCACACCATAAGCAT	59	0.8	5	248–270	6-Fam	Olsen et al. 1998
	R: GTTTCTTCGACTGTTTCCTCTGTGTTGAG						
*Omm-1032*	F: GCGAGGAAGAGAAAGTAGTAG	57	0.8	7	203–227	Ned	You-Yi et al. 2009
	R: CCCATCTTCTCTCTGATTATG						
*Omm-1077*	F: GGCTGACCAGAGAAAGACTAGTTC	60	1.0	26	325–467	Pet	You-Yi et al. 2009
	R: TGTTACGGTGTCTGACATGC						
*Omm-1088*	F: CTACAGGCCAACACTACAATC	61	0.8	6	112–159	Pet	You-Yi et al. 2009
	R: CTATAAAGGGAATAGGCACCT						
*Sfo-18*	F: TGGTGTATCCTGCTCCTG	56	0.8	4	163–207	6-Fam	Geist et al. 2009
	R: TGGAATGTGTGTCTGTTTTCT						
*Sfo-262*	F: CCCATGTCAGTATTGGACTC	60	0.8	3	186–228	Ned	Perry et al. 2005
	R: CTTCATGGGCAGAATGGAC						
*Ssa-197*	F: GGGTTGAGTAGGGAGGCTTG	59	0.8	8	132–180	Vic	O’Reilly et al. 1996
	R: TGGCAGGGATTTGACATAAC						

### Genotyping

Genotyping of microsatellite DNA fragments was conducted using an Applied Biosystems 3130 Genetic Analyzer. In order to enable genotyping of PCR products forward primers were labeled with different fluorescent reporter dyes (PET-red, VIC-green, 6-FAM-blue and NED-yellow) (Table 1). The GeneScan 600 LIZ size standard was utilized as a reference for determining the length of examined DNA fragments. Individual microsatellite loci amplified using primers with different attached fluorescent dyes were arranged into sets and analyzed in multiplex mode. In order to visualize the results, software provided by manufacturer Genemaper v4.1 software and Data Collection Software v3.0 (Applied Biosystems, California, USA) were used. The genetic profiles containing the list of alleles detected within the studied loci were prepared for each fish.

### Data analysis

The Micro-Checker software (version 2.2.3) was used to check microsatellite null alleles, scoring errors due to stuttering and large allele drop-out in samples (Van Oosterhout et al. 2004). The observed number of alleles per locus, allele frequency, number of private alleles, allelic range and allelic richness (*A*_*r*_) were computed by GenePop software (version 4.2.1) (Rousset 2008). The observed (*Ho*) and expected heterozygosity (*He*), the exact Hardy-Weinberg (H-W) equilibrium test, as well as linkage disequilibrium (LD) were calculated using Arlequin software (version 3.5) (Excoffier and Lischer 2010). Each locus and each broodstock was tested separately. The genetic differences between the sampled broodstocks and fixation index (*Fis*) were calculated using Fstat software (version 2.9.3) (Goudet 2001). The polymorphism information content (*PIC* value) was also calculated employing PowerMarker software (version 3.25) (Liu and Muse 2005). Genetic divergence between studied broodstocks of European huchen was analyzed using two different parameters: genetic differentiation index (*Fst*) and Nei’s genetic distance. Shannon’s index (*I*) for each loci within tested broodstocks was calculated using PopGene software (version 1.3.2) (Yeh and Boylet 1997). The UPGMA dendrogram based on Nei’s genetic distance was constructed by MEGA6 (version 6.0.5) (Tamura et al. 2013). An analysis of molecular variance (AMOVA) was done with the Arlequin package 3.5 for measuring variance within and between broodstocks. The likely occurrence of bottleneck or the founder effect and their influence on within-broodstock genetic variability was based on the Garza-Williamson index (*M*), which was computed applying Arlequin software 3.5. A test for bottleneck assessment was also conducted using the Bottleneck software (version 1.9) (Piry et al. 1999), which tests for departure from mutation drift equilibrium based on heterozygosity excess or deficiency. Recent broodstock bottlenecks assuming a stepwise mutation model (*SMM*) and infinite allele model (*IAM*) for four broodstocks of European huchen were tested. This method is based on the assumption that in non-bottlenecked broodstock (close to mutation drift equilibrium) the value of expected heterozygosity (*He*) is equal to *Heq* (heterozygosity expected in a mutation-drift equilibrium). The excess of *He* over *Heq* is the evidence of severe reduction in broodstock effective size that may occur because of a bottleneck event. In order to accommodate the obtained genotypic data to the requirements of employed software, every tetrasomic locus was examined as two disomic loci and as result the mean values was considered for estimation of genetic parameters.

## Results

In the present study the 29 microsatellite DNA fragments were applied to evaluate the genetic diversity in four broodstocks of European huchen. Among these 29 microsatellites, 11 (*Str-12*, *Str-15*, *Str-60*, *Str-73*, *Str-543*, *Ssa-85*, *Ssa-171*, *BleTet-2*, *Sfo-292*, *Omm-1016*, *Omm-5017*) were monomorphic in all broodstocks, one (*Str-85*) did not produce any PCR product and seven loci (*Hljz-003*, *Hljz-023*, *Hljz-031*, *Hljz-069*, *Omm-1097*, *Omm-1125*, *Omm-5000*) produced non specified bands (stutter bands). Ten microsatellite loci (*BleTet-9*, *BleTri-2*, *Hljz-056*, *Ogo-2*, *Omm-1032, Omm-1077*, *Omm-1088*, *Sfo-*18, *Sfo-*262, *Ssa-197*) were selected for further analyses because they were characterized by good quality products and were polymorphic (Table 1). Among these, loci: *BleTet-9*, *BleTri-2*, *Hljz-056*, *Ogo-2*, *Omm-1032*, *Omm-1088*, *Sfo-18*, *Ssa-197* were considered as disomic and *Omm-1077*, *Sfo-262* were tetrasomic. The size of the alleles in an individual locus varied between 112 base pairs (bp) and 467 bp. The number of amplified alleles per locus ranged from one (*Sfo-262*) to 26 (*Omm-1077*) with an average of 8.1 alleles per locus (Table 1).

The examined broodstocks of European huchen differed in the number of alleles detected in locus as well as in the overall number of alleles identified across all investigated loci. Moreover, the allelic frequency distribution for a number of loci was quite different among the four studied broodstocks. The null alleles were detected in German and Slovak broodstocks, which appeared in *Omm-1077* loci at 0.086 and 0.063 frequencies, respectively (Table 2).

**Table 2:** Allele frequencies at microsatellite loci in studied European huchen broodstocks. Null: null allele

Locus	Alleles	Allele frequencies by locus			
		Poland	Germany	Slovakia	Ukraine
*Sfo-262*	186	1.000	0.516	0.693	1.000
	220	0.000	0.445	0.216	0.000
	228	0.000	0.039	0.091	0.000
*Ssa-197*	132	0.000	0.000	0.091	0.000
	136	0.000	0.109	0.000	0.000
	140	0.000	0.234	0.011	0.000
	144	0.000	0.000	0.091	0.000
	148	0.933	0.500	0.295	0.948
	156	0.017	0.094	0.091	0.000
	160	0.000	0.000	0.136	0.000
	180	0.050	0.063	0.284	0.052
*Sfo-18*	163	0.000	0.078	0.000	0.000
	168	0.433	0.609	0.273	0.466
	194	0.333	0.078	0.648	0.241
	207	0.233	0.234	0.080	0.293
*Ogo-2*	248	0.000	0.031	0.000	0.000
	256	0.000	0.203	0.148	0.000
	258	0.400	0.484	0.261	0.241
	266	0.600	0.281	0.591	0.724
	270	0.000	0.000	0.000	0.034
*BleTri-2*	118	0.000	0.234	0.080	0.034
	124	0.050	0.000	0.000	0.017
	127	0.950	0.766	0.920	0.948
*BleTet-9*	281	0.000	0.094	0.000	0.000
	292	0.000	0.172	0.000	0.000
	304	0.183	0.000	0.000	0.517
	308	0.200	0.188	0.068	0.052
	312	0.033	0.031	0.000	0.000
	337	0.000	0.016	0.170	0.000
	341	0.000	0.000	0.011	0.000
	353	0.417	0.375	0.091	0.207
	357	0.083	0.047	0.000	0.190
	365	0.033	0.078	0.136	0.000
	390	0.000	0.000	0.136	0.000
	394	0.033	0.000	0.000	0.000
	410	0.017	0.000	0.000	0.034
	418	0.000	0.000	0.375	0.000
	422	0.000	0.000	0.011	0.000
*Hljz-056*	224	0.000	0.047	0.000	0.000
	230	0.117	0.063	0.114	0.138
	233	0.183	0.344	0.000	0.138
	236	0.700	0.547	0.886	0.724
*Omm-1032*	203	0.283	0.000	0.227	0.276
	205	0.000	0.234	0.080	0.000
	209	0.067	0.078	0.000	0.259
	211	0.483	0.406	0.693	0.448
	215	0.000	0.063	0.000	0.000
	225	0.167	0.000	0.000	0.017
	227	0.000	0.219	0.000	0.000
*Omm-1088*	112	0.133	0.297	0.409	0.069
	116	0.417	0.109	0.136	0.500
	120	0.267	0.438	0.261	0.138
	151	0.000	0.000	0.000	0.017
	155	0.183	0.031	0.182	0.276
	159	0.000	0.125	0.011	0.000
*Omm-1077*	325	0.100	0.023	0.000	0.112
	332	0.233	0.078	0.227	0.129
	336	0.192	0.148	0.000	0.276
	340	0.000	0.008	0.000	0.000
	346	0.017	0.000	0.000	0.000
	349	0.000	0.008	0.000	0.000
	353	0.025	0.188	0.222	0.000
	355	0.000	0.008	0.000	0.000
	359	0.008	0.000	0.000	0.000
	361	0.100	0.039	0.000	0.121
	363	0.000	0.086	0.182	0.000
	365	0.008	0.000	0.000	0.009
	371	0.008	0.000	0.000	0.060
	375	0.017	0.000	0.000	0.052
	378	0.000	0.031	0.006	0.000
	382	0.033	0.000	0.034	0.026
	386	0.167	0.102	0.051	0.112
	402	0.000	0.016	0.000	0.000
	406	0.083	0.039	0.045	0.103
	416	0.000	0.016	0.000	0.000
	418	0.008	0.000	0.000	0.000
	447	0.000	0.000	0.023	0.000
	451	0.000	0.086	0.057	0.000
	455	0.000	0.000	0.091	0.000
	467	0.000	0.039	0.000	0.000
	null	0.000	0.086	0.063	0.000

One microsatellite locus, *Sfo-262*, was monomorphic in Polish and Ukrainian broodstocks (Table 2). Eight microsatellite loci, *BleTet-9*, *Hljz-056*, *Ogo-2*, *Omm-1032*, *Omm-1077*, *Omm-1088*, *Sfo-18*, and *Ssa-197*, were highly polymorphic in the groups of fish studied (Table 2). The high degree of polymorphism of studied loci implies that each locus is informative and could be used in population studies. The genetic diversity parameters (*Ho*, *He*, *A*_*o*_, *A*_*e*_, *A*_*r*_, *I*, and *PIC*) of the four broodstocks of European huchen are shown in Tables 3 and 4. The mean values of the polymorphism information content (*PIC*) in fish from Poland, Germany, Slovakia, and Ukraine were 0.430, 0.590, 0.496, and 0.405, respectively. The mean allelic richness varied from 3.944 to 5.200 in studied broodstocks of European huchen (Table 3). Private alleles were identified in all analyzed broodstocks (Table 3). The German broodstock was characterized by the highest number of the private alleles (15) in the studied microsatellite loci.

**Table 3:** Genetic diversity parameters of four European huchen broodstocks analyzed. *A*_r_: allelic richness, *A*_o_: observed alleles, *A*_e_: expected alleles, *I*: Shannon’s index, *PIC*: polymorphism information content, MONO: monomorphic locus

Broodstock	Locus	*A*_r_	*A*_o_	*A*_e_	*I*	*PIC*	Private alleles
Poland	*Sfo-262*	1.000	1	MONO	0.0000	0.000	–
	*Ssa-197*	2.967	3	1.1443	0.2824	0.121	–
	*Sfo-18*	3.000	3	2.8302	1.0681	0.572	–
	*Ogo-2*	2.000	2	1.9231	0.6730	0.365	–
	*BleTri-2*	2.000	2	1.1050	0.1985	0.090	–
	*BleTet-9*	7.965	8	3.8793	1.6131	0.709	394
	*Hljz-056*	3.000	3	1.8614	0.8113	0.416	–
	*Omm-1032*	4.000	4	2.8892	1.1879	0.596	–
	*Omm-1088*	4.000	4	3.3771	1.2969	0.653	–
	*Omm-1077*	11.349	14	5.3445	1.9050	0.777	346, 359, 418
	Mean	4.128	4.4	2.7060	0.9118	0.430	
Germany	*Sfo-262*	2.953	3	2.1458	0.8203	0.426	–
	*Ssa-197*	5.000	5	3.0341	1.3239	0.627	136
	*Sfo-18*	4.000	4	2.2806	1.0402	0.510	163
	*Ogo-2*	3.993	4	2.8093	1.1400	0.580	248
	*BleTri-2*	2.000	2	1.5598	0.5445	0.294	–
	*BleTet-9*	7.898	8	4.4716	1.7222	0.748	281, 292
	*Hljz-056*	3.999	4	2.3622	1.0139	0.501	224
	*Omm-1032*	5.000	5	3.5993	1.4109	0.678	215, 227
	*Omm-1088*	4.993	5	3.2456	1.3325	0.642	–
	*Omm-1077*	12.167	17	7.6171	2.1939	0.845	340, 349, 353, 355, 402, 416, 467
	Mean	5.200	5.6	3.3125	1.2542	0.590	
Slovakia	*Sfo-262*	2.998	3	1.8602	0.7937	0.407	–
	*Ssa-197*	6.659	7	4.7277	1.6943	0.756	132, 144, 160
	*Sfo-18*	3.000	3	1.9990	0.8370	0.431	–
	*Ogo-2*	3.000	3	2.2763	0.9441	0.495	–
	*BleTri-2*	2.000	2	1.1716	0.2777	0.136	–
	*BleTet-9*	7.317	8	4.5446	1.7156	0.753	341, 390, 418, 422
	*Hljz-056*	2.000	2	1.2523	0.3541	0.181	–
	*Omm-1032*	3.000	3	1.8571	0.7921	0.405	–
	*Omm-1088*	4.659	5	3.4789	1.3489	0.664	–
	*Omm-1077*	7.589	11	5.7025	1.7131	0.733	378, 447
	Mean	4.222	4.7	2.8870	1.0470	0.496	
Ukraine	*Sfo-262*	1.000	1	MONO	0.0000	0.000	–
	*Ssa-197*	2.000	2	1.1088	0.2036	0.093	–
	*Sfo-18*	3.000	3	2.7710	1.0587	0.567	–
	*Ogo-2*	3.000	3	1.7128	0.6929	0.354	270
	*BleTri-2*	3.000	3	1.1102	0.2365	0.097	–
	*BleTet-9*	5.000	5	2.8557	1.2516	0.602	–
	*Hljz-056*	3.000	3	1.7780	0.7802	0.397	–
	*Omm-1032*	4.000	4	2.9050	1.1347	0.588	–
	*Omm-1088*	5.000	5	2.8557	1.2295	0.596	151
	*Omm-1077*	7.500	10	5.0937	1.7251	0.755	–
	Mean	3.944	4.22	2.4656	0.831	0.405

**Table 4:** Comparison of observed (*Ho*) and expected (*He*) heterozygosity, expected heterozygosity (*Heq*) in an infinite allele model (*IAM*) and stepwise mutation model (*SMM*), Garza-Williamson index (*M*) and fixation index (*Fis*) in studied European huchen boodstocks: P-level of significance, MONO: monomorphic locus. Deviations statistically significant at *P* < 0.05

Broodstock/locus	*Ho*	*He*	*P*	*IAM*		*SMM*		*Fis*	*M*
				*Heq*	*P*	*Heq*	*P*		
Poland									
*Sfo-262*	MONO	0.000	–	–	–	–	–	–	1.000
*Ssa-197*	0.133	0.128	1.000	0.344	0.164	0.476	0.018	−0.040	0.091
*Sfo-18*	0.800	0.658	0.324	0.364	0.024	0.473	0.041	−0.221	0.075
*Ogo-2*	0.467	0.488	1.000	0.205	0.080	0.250	0.127	0.045	0.222
*BleTri-2*	0.100	0.097	1.000	0.202	0.427	0.256	0.287	−0.036	0.500
*BleTet-9*	0.667	0.755	0.045	0.709	0.404	0.813	0.082	0.119	0.075
*Hljz-056*	0.467	0.471	0.883	0.358	0.353	0.476	0.381	0.009	0.429
*Omm-1032*	0.767	0.665	0.197	0.468	0.083	0.608	0.329	−0.156	0.174
*Omm-1088*	0.800	0.716	0.662	0.462	0.021	0.604	0.077	−0.120	0.091
*Omm-1077*	1.000	0.816	0.074	0.812	0.433	0.879	0.020	−0.241	0.135
Mean	0.520	0.478	0.576	0.436	0.221	0.537	0.151	−0.071	0.279
Germany									
*Sfo-262*	0.969	0.542	0.000	0.356	0.167	0.471	0.344	−0.814	0.070
*Ssa-197*	0.687	0.681	0.757	0.539	0.180	0.682	0.409	−0.010	0.111
*Sfo-18*	0.594	0.570	1.000	0.459	0.310	0.598	0.338	−0.042	0.089
*Ogo-2*	0.750	0.654	0.351	0.463	0.108	0.602	0.380	−0.149	0.210
*BleTri-2*	0.406	0.365	0.653	0.216	0.276	0.246	0.329	−0.116	0.200
*BleTet-9*	0.594	0.789	0.007	0.701	0.217	0.810	0.248	0.250	0.094
*Hljz-056*	0.625	0.586	0.745	0.458	0.264	0.595	0.372	−0.068	0.308
*Omm-1032*	0.750	0.734	0.884	0.550	0.065	0.684	0.304	−0.023	0.217
*Omm-1088*	0.562	0.703	0.034	0.539	0.143	0.677	0.470	0.202	0.104
*Omm-1077*	0.922	0.876	0.002	0.819	0.148	0.886	0.221	−0.055	0.058
Mean	0.686	0.650	0.443	0.510	0.188	0.625	0.342	−0.082	0.146
Slovakia									
*Sfo-262*	0.614	0.468	0.036	0.335	0.310	0.464	0.407	−0.317	0.070
*Ssa-197*	0.954	0.797	0.000	0.636	0.050	0.771	0.363	−0.200	0.143
*Sfo-18*	0.523	0.505	0.320	0.330	0.211	0.458	0.442	−0.035	0.075
*Ogo-2*	0.682	0.567	0.264	0.330	0.098	0.460	0.229	−0.205	0.273
*BleTri-2*	0.159	0.148	1.000	0.188	0.491	0.233	0.425	−0.075	0.200
*BleTet-9*	0.659	0.789	0.003	0.674	0.138	0.803	0.309	0.166	0.070
*Hljz-056*	0.182	0.204	0.437	0.193	0.399	0.242	0.497	0.109	0.286
*Omm-1032*	0.250	0.467	0.000	0.337	0.314	0.464	0.412	0.467	0.333
*Omm-1088*	0.818	0.721	0.086	0.524	0.064	0.668	0.292	−0.137	0.104
*Omm-1077*	0.966	0.785	0.000	0.645	0.088	0.764	0.256	−0.246	0.033
Mean	0.581	0.545	0.215	0.419	0.216	0.533	0.363	−0.047	0.159
Ukraine									
*Sfo-262*	MONO	0.000	–	–	–	–	–		1.000
*Ssa-197*	0.103	0.100	1.000	0.210	0.424	0.246	0.314	−0.037	0.061
*Sfo-18*	0.690	0.650	0.383	0.361	0.024	0.479	0.060	−0.062	0.075
*Ogo-2*	0.241	0.423	0.030	0.354	0.419	0.473	0.310	0.434	0.231
*BleTri-2*	0.103	0.101	1.000	0.354	0.124	0.473	0.013	−0.024	0.300
*BleTet-9*	0.552	0.661	0.034	0.557	0.298	0.685	0.295	0.168	0.047
*Hljz-056*	0.483	0.445	0.533	0.363	0.404	0.479	0.323	−0.086	0.429
*Omm-1032*	0.655	0.667	0.521	0.465	0.101	0.605	0.290	0.018	0.174
*Omm-1088*	0.552	0.661	0.086	0.551	0.274	0.686	0.311	0.168	0.114
*Omm-1077*	0.965	0.817	0.006	0.711	0.091	0.810	0.394	−0.100	0.116
Mean	0.434	0.452	0.399	0.4362	0.240	0.548	0.257	0.053	0.255

The mean observed heterozygosity (*Ho*) in the investigated broodstocks ranged from 0.434 (Ukraine) to 0.686 (Germany) and were close to the mean values expected under H-W equilibrium (*He*) (Table 4). Slovak broodstock had the highest level of deviation (five loci) and three of these loci exhibited heterozygote excess (Table 4). Polish broodstock had the lowest deviation (one loci with heterozygote deficiency). Table 4 also shows expected heterozygosity in two models of a mutation-drift equilibrium (*Heq*). In all broodstocks, under the infinite allele model (*IAM*) and stepwise mutation (*SMM*) models, heterozygosity excess were detected in most of analyzed loci; however, observed *He*>*Heq *differences were significant in only Polish (two out of ten loci were analyzed) and Ukrainian (one out of ten loci were analyzed) broodstocks. All investigated loci differed in terms of the Garza-Williamson index (*M*). In all samples *M* values were the lowest for the most polymorphic loci (such as *BleTet-9* and *Omm-1077*) and the highest for the least polymorphic loci (*BleTri-2, Hljz-056*). The mean observed *M* values in the examined broodstocks varied from 0.146 to 0.279. The mean fixation index (*Fis*) in fish from Poland, Germany, Slovakia, and Ukraine were −0.071, −0.082, −0.047, and 0.053, respectively (Table 4).

**Fig. 2:**
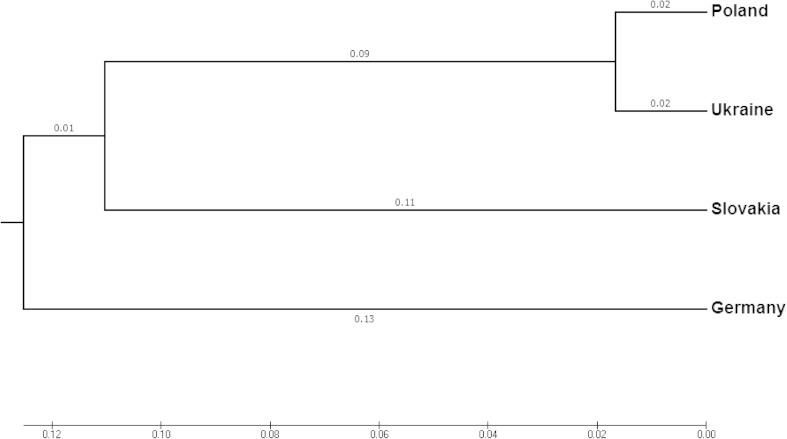
UPGMA dendrogram based on Nei’s genetic distance illustrating relationships among four European huchen broodstocks under current study

The genetic differentiation (*Fst*) of the four broodstocks of European huchen was 0.1139. Analysis of the genetic structure of the studied broodstocks with AMOVA method revealed that 13.20 % of the genetic diversity was distributed among broodstocks and 86.80 % occurred among individuals within the broodstocks. The Nei’s genetic distances among the four broodstocks and the Fst matrix are shown in Table 5. The highest genetic divergence, calculated as *Fst* values, occurred between Ukraine and Slovakia (0.1731). The lowest genetic divergence and genetic distance were observed between Poland and Ukraine (0.0234 and 0.0333, respectively), while the highest genetic distances were observed among German, Slovak, and Ukrainian broodstocks (Fig. 2).

**Table 5:** Nei’s genetic distance (below diagonal) and *Fst* (above diagonal) of four European huchen broodstocks analyzed

Broodstock	Poland	Germany	Slovakia	Ukraine
Poland		0.1361	0.1361	0.0234
Germany	0.2226		0.1192	0.1711
Slovakia	0.1928	0.2427		0.1731
Ukraine	0.0333	0.2857	0.2483

## Discussion

Microsatellite DNA analysis is a powerful tool for the monitoring of restocking programs. Such molecular based screening is essential for understanding population genetic differentiation among reared stocks within species, inferring parentage in mixed-family assemblages, maintaining genetic variability in populations, estimating the effective size of populations as well as inferring the effects of selection within raised stocks (Vrijenhoek 1998; Frankham et al. 2002; Hellerman et al. 2007). In the present study, microsatellite DNA analysis technique successfully applied for the first time in the Polish broodstock of European huchen provided new information about genetic structure of this valuable salmonid fish species.

The comparison of the overall number of private alleles, allelic ranges and its frequency clearly demonstrates genetic differences between studied broodstocks of European huchen. This confirms the hypothesis that the broodstocks studied do not share a common gene pool at the population genetic level. Genetic diversity, which can be evaluated as the allele number by locus and the mean number across loci, differed among the studied groups of fish. Examined broodstocks of European huchen exhibited moderate genetic diversity (the mean *He* and *PIC* per broodstock were between 0.4–0.5) with the exception of German broodstock which presented high genetic diversity (the mean *He* and *PIC* were close to 0.6). Similar moderate indices of genetic diversity to those observed in the present study were reported for some populations of taimen from Heilongjiang River Basin (China) (Guangxiang et al. 2006; You-Yi et al. 2009; Liu et al. 2011) and for the Huchen populations from Europe (Geist et al. 2009; Weiss et al. 2011), evidencing that our results are comparable to those described in other studies (Liang et al. 2004; Froufe et al. 2004). All genetic diversity parameters (*Ho*, *He*, *A*_*o*_, *A*_*e*_, *A*_*r*_, *I*, and *PIC*) consistently ranked the broodstocks in order of decreasing diversity (Germany > Slovakia > Poland > Ukraine). However, there were no significant differences between these parameters among the four studied broodstocks (*P* > 0.05).

According to the current results all analyzed broodstocks are in H-W equilibrium. However, from 8 to 42 % of the studied loci deviated from H-W equilibrium, suggesting that both the genotype and gene frequencies fluctuated continuously as a response to the different stocking conditions. The average *Fis* value was positive only in Ukrainian broodstock (0.053) indicating a small overall deficiency of heterozygotes, while the rest of the analyzed broodstocks of European huchen were characterized by negative *Fis* indicators exhibiting small overall excess of heterozygotes against Hardy-Weinberg expectations. In contrast, in domesticated stocks of fish the observed heterozygosity (*Ho*) sometimes exceeds the expected heterozygosity (*He*). For example, this tendency was observed in Chinook salmon (*Oncorhynchus tshawytscha*) stocks (Kim et al. 2004) and paddlefish (*Polyodon spathula*) (Kaczmarczyk et al. 2012). This excess may be a consequence of the use of a non-random subset of the broodstock in the hatchery conditions (Luikart and Cornuet 1998; 1999). The bottleneck test applied in the present study showed evidence of a bottleneck under the infinite allele (*IAM*) and stepwise mutation models (*SMM*) in Polish and Ukrainian broodstocks. The heterozygosity excess at observed loci in mentioned broodstocks might be indicative of a small founding population size or bottleneck events as was hypothesized in the case of the hatchery stocks of brook trout (*Salvelinus fontinalis*) (Fopp-Bayat et al. 2010) and paddlefish (Kaczmarczyk et al. 2012). In the present study the average values of the Garza-Williamson index for every examined broodstock were surprisingly low (0.146–0.279) suggesting that all investigated broodstocks suffered from bottleneck or founder effects in the past (Garza and Williamson 2001; Tzika et al. 2008; Kaczmarczyk and Zuchowska 2011). The historical information together with genetic data on Polish broodstock of European huchen confirm the occurrence of the bottleneck effect in the past.

Our results showed that some genetic diversity among the broodstocks occurred (13.20 %); however, the majority of diversity were observed between individuals within a broodstocks (86.80 %). Similar genetic diversity (13.10 %) was observed among two hatchery stocks of barfin flounder (*Verasper moseri*) from China (Hongyu et al. 2009). Lower genetic diversity were reported between four taimen populations from Heilongjiang River Basin (6.12 %) (You-Yi et al. 2009). These presented data suggest that there is substantial genetic differentiation between tested broodstocks of European huchen. A low level (0.023) of genetic differentiation was detected between Polish and Ukrainian broodstocks. This implies that these broodstocks have a similar genetic structure. Contrastingly, the genetic distances observed between German, Slovak, and Ukrainian broodstocks were moderate because the rate of genetic distance of these groups of fish was 0.1731 (Balloux and Lugon-Moulin 2002). Numerous examples evidencing that mixed-source reintroductions by genetically distant populations may result in outbreeding depression (Gharrett et al. 1999; Alacs et al. 2007; Huff et al. 2011). Thus, any enrichment to the genetic pool of a conserved population should be done with genetically similar material. According to the present results, if a supplement of the genetic pool of Polish broodstock of European huchen will be necessary in the future, the Ukrainian broodstock seems to be the most suitable material to maintain the genetic diversity of this stock.

A continuous genetic monitoring of hatchery stocks is an important tool for development of sustainable conservation management programs. Management based on supportive and captive breeding carries the risk of adverse modifications to a gene pool, resulting in loss of genetic diversity, decrease of heterozygosity, or inbreeding depression. The consequence of inbreeding depression in hatchery stocks can be the negative effects on: hatching rate, fry survival, growth rate, and spawning performance, as well as inefficiency of feed conversion, occurrence of deformations and presence of short-lived albino fish (Guo-Sheng et al. 1996; Pante et al. 2001; Wang et al. 2002; Ala-Honkola et al. 2009). Supportive breeding systems are complex and the long-term genetic consequences are unclear and difficult to predict (Duchesne and Bernatchez 2002). For example, the common practice of mixed-milt fertilization, systematic selection of specified phenotypes and feeding of salmonid fish with trout pellets under hatchery conditions may lead to selection of genotypes that have lower viability in the wild (Flagg et al. 1995; Waples 1999; Glover et al. 2001, 2004; Snook 2005; Wedekind et al. 2007). The efficacy of artificial stocking in order to maintain natural populations is one of the most controversial topics in fishery management. It is considered that the post-release survival rate of reared fish is essentially lower than that of wild fish (Hoekstra et al. 2007; Araki et al. 2008). The long-term assessment of results of historic stocking of salmonids in Europe, including European huchen are very poorly known. Thus, the effects of hatchery fish on wild populations remain an open question and a topic of major concern. In the case of European huchen, stocking activities have not been well documented and no official information is therefore available regarding the purpose of stocking and the origin or the numbers of stocked fish.

The potential for evolution and adaptation to new environments is limited by the genetic diversity of the population. Natural selection favors individuals that are better adapted to live in natural environment than in captivity, thus research programs aimed at retaining the evolutionary genetic adaptive potential of European huchen stocks are very important. Therefore the optimum degree of genetic differentiation and a high genetic diversity should be permanently controlled. Moreover, European huchen habitat continues to be threatened by further anthropogenic factors influence and particularly unfavorable conditions may adversely impact on the populations characterized by low genetic variation level. The lack of information on the genetic structure of European huchen broodstock in Poland essentially limits the sustainable conservation of this species. Therefore, baseline genetic data are crucial to guide future population specific conservation programs and research efforts on European huchen in Poland.
